# To Our Readers

**Published:** 1881-12

**Authors:** 


### HALL'S JOURNAL OF HEALTH

AND

### MISCELLANY.

MONTHLY.

E. H. GIBBS, A. M., M. D., EDITOR.

FOUNDED IN 1854.

### TO OUR READERS.

MOST of our subscriptions end with this December number of the JOURNAL OF HEALTH.

It has not been our custom to give special notice to each subscriber when his subscription ends, nor to continue to send the JOURNAL beyond the time for which it has been paid. Those who properly appreciate its teachings miss it when it ceases to come and promptly send for it, and those who do not prize health until they lose it usually prefer a different style of literature.

This number completes the 28th year of HALL'S JOURNAL OF HEALTH. It is the oldest publication of the kind in the world, and is doubtless the best. Its founder—the late Dr. W. W. Hall—was the most popular writer on health subjects that has lived in this century or in any other. It would be impossible to overestimate the amount of good he did through the JOURNAL OF HEALTH, in educating the people to right methods of living. We have endeavored to continue the work much after the plan instituted by him, and the prosperity of the JOURNAL OF HEALTH, which has enabled us to enlarge it and to improve its appearance, is evidence that our efforts have been duly appreciated.

We print in this number an index for the year for the convenience of many of our subscribers who have their numbers bound. We ask our readers to glance over this index and then determine if as much useful information is elsewhere to be found for two dollars a year as is contained in HALL'S JOURNAL OF HEALTH.

